# CKD Stages, Bone Metabolism Markers, and Cortical Porosity Index: Associations and Mediation Effects Analysis

**DOI:** 10.3389/fendo.2021.775066

**Published:** 2021-11-05

**Authors:** Yan Xiong, Tongxiang He, Yanan Wang, Weiyin Vivian Liu, Shuang Hu, Yao Zhang, Donglin Wen, Bowen Hou, Yitong Li, Peisen Zhang, Jianyi Liu, Fan He, Xiaoming Li

**Affiliations:** ^1^ Department of Radiology, Tongji Hospital, Tongji Medical College, Huazhong University of Science and Technology, Wuhan, China; ^2^ MR Research, GE Healthcare, Beijing, China; ^3^ Department of Rehabilitation Medicine, School of Medicine, Guangzhou First People’s Hospital, South China University of Technology, Guangzhou, China; ^4^ Renhe Shijia, Wuhan, China; ^5^ Department of Nephrology, Tongji Hospital, Tongji Medical College, Huazhong University of Science and Technology, Wuhan, China

**Keywords:** chronic kidney disease, CKD-MBD, Ultrashort TE, porosity index, cortical porosity, mediation analysis, bone metabolism markers

## Abstract

Chronic kidney disease (CKD) has a significant negative impact on bone health. However, the mechanisms of cortical bone deterioration and cortical porosity enlargement caused by CKD have not been fully described. We therefore examined the association of CKD stages with cortical porosity index (PI), and explored potential mediators of this association. Double-echo ultrashort echo-time magnetic resonance imaging (UTE MRI) provides the possibility of quantifying cortical porosity *in vivo*. A total of 95 patients with CKD stages 2-5 underwent 3D double-echo UTE-Cones MRI (3.0T) of the midshaft tibia to obtain the PI. PI was defined as the ratio of the image signal intensity of a sufficiently long echo time (TE) to the shortest achievable TE. Parathyroid hormone (PTH), β-CrossLaps (β-CTX), total procollagen type I amino-terminal propeptide (T-P1NP), osteocalcin (OC), 25-hydroxyvitamin D (25OHD), and lumbar bone mineral density (BMD) were measured within one week of the MRI. Partial correlation analysis was performed to address associations between PI, eGFR and potential mediators (PTH, β-CTX, T-P1NP, OC, 25OHD, BMD, and T-score). Multiple linear regression models were used to assess the association between CKD stages and PI value. Then, a separate exploratory mediation analysis was carried out to explore the impact of CKD stages and mediators on the PI value. The increasing CKD stages were associated with a higher PI value (P_trend_ < 0.001). The association of CKD stages and PI mediated 34.4% and 30.8% of the total effect by increased PTH and β-CTX, respectively. Our study provides a new idea to monitor bone health in patients with CKD, and reveals the internal mechanism of bone deterioration caused by CKD to some extent.

## Introduction

Chronic kidney disease (CKD) has become a recognized global public health problem, which affects approximately 0.75 billion human beings worldwide (1). CKD has a significant negative impact on bone health (2, 3), which results in a two-fourfold higher risk of fracture in CKD patients compared with general population, and the occurrence of fractures is associated with increased mortality (4, 5). Cortical bone is the main factor that determines the overall bone strength, accounting for more than 80% of the total bone (6). Cortical porosity is the main factor that determines the strength of cortical bone, and the development of cortical pores leads to an increase in bone fragility, which increases the fracture (7). A study by Nickolas et al. (8) found that hemodialysis has an independent effect on the increase in cortical porosity, which suggests that cortical porosity may increase as CKD progresses.

CKD-mineral and bone disorder (CKD-MBD), a disease of bone metabolism that manifests as systemic mineral and bone metabolism disorders, is the main complication of CKD and affects most patients with moderate to severe CKD (9). Elevated parathyroid hormone (PTH) is a potential mediator for patients with MBD, and it is also the most widely studied and most recognized bone metabolism marker for predicting bone turnover in CKD patients (10). In terms of the mechanism, with the progression of CKD and calcium and phosphorus metabolism disorders, patients develop secondary hyperparathyroidism to varying degrees, and increased PTH has a catabolic effect on cortical bone (11). Cortical porosity in patients with CKD showed highly positive correlation with PTH (r = 0.62; P = 0.021) (3). In addition, tartrate-resistant acid phosphatase 5b (TRAP5b) and bone-specific alkaline phosphatase (BSAP) are less affected by renal clearance and can more accurately assess MBD (12), but these two indicators are not as widely used in clinical practice as PTH. The application of other bone metabolism markers (e.g., β-CrossLaps (β-CTX), total procollagen type I amino-terminal propeptide (T-P1NP), osteocalcin (OC), and 25-hydroxyvitamin D (25OHD), etc.) in CKD is controversial, and there are few related studies. These bone metabolism markers are relatively common in the study of osteoporosis. In this study, we wanted to explore which metabolic markers influenced the relationship between CKD and cortical porosity, and therefore some of these metabolic markers were included.

Early imaging studies on CKD-MBD mainly focused on trabecular bone (13). However, recent research has shown that cortical bone plays a more crucial part than trabecular bone in the bone health of CKD-MBD patients (14). In patients with CKD, the loss of cortical bone is associated with increased cortical porosity and decreased cortical thickness (8). Nevertheless, cortical bone is not taken as a key parameter in the “turnover, mineralization, volume” classification of CKD-MBD (3).

At present, information on the porosity of human cortical bone *in vivo* is mainly obtained through high-resolution peripheral quantitative computed tomography (HR-pQCT) and bone biopsy (15). However, HR-pQCT involves radiation, and its limited resolution underestimates porosity (16). And bone biopsy is expensive and invasive and requires professional knowledge to perform, process, and interpret; thus, only a few laboratories worldwide carry out this test (15). With the development of magnetic resonance technology, the adoption of ultrashort echo time (UTE) *in vivo* has proven to be feasible for quantitative assessment of cortical pore information (17). Magnetic resonance quantification of bone tissue has always been a difficult problem in the field of magnetic resonance research. UTE magnetic resonance imaging (MRI) is an advanced and promising method. Its echo time is very short, which solves the difficult problem of the inability of conventional MRI sequences to obtain bone signals. At present, this sequence has been used for the quantitative and qualitative assessment of cortical bone pore water (e.g., bicomponent analysis, tricomponent analysis, single/double adiabatic inversion of UTE technology, etc.) (18), and increased pore water content has been confirmed to be related to increase in cortical porosity and decrease in mechanical properties (19). However, these methods are complicated, the scanning time is very long, and the postprocessing technology requirements are very strict, so they are hardly used in clinical research. Rajapakse et al. (20) evaluated cortical bone porosity using a novel double-echo UTE MR sequence recently. The porosity index (PI) was defined as the ratio of the image signal intensity of a sufficiently long echo time (TE) (mainly from the pore water signal) to the shortest achievable TE (from all water signals), representing the fraction of pore-water. The selection of TE value needs to consider the T2* value of bound water (0.3-0.4 ms) and pore water (> 1 ms) (20, 21). The researchers found that the PI measured by this method exhibits highly positive correlation with cortical porosity measured by micro-CT (R^2^ = 0.79). Furthermore, this means showed high repeatability when measuring vivo tibial PI (with a variation of 2.2% in between-day coefficient). Thus, it is reasonable for us to conclude that PI can reflect porosity to a large extent. This method, which is noninvasive and does not involve radiation, has a short scanning time and a simple processing protocol, which results in a high potential for application in clinical practice. Chen et al. (22) applied this method to healthy volunteers. However, there are almost no relevant studies on applying this method to various metabolic bone diseases, including CKD-MBD.

The mediators selected in this study are currently the most recognized mediators in bone metabolism, and their application in CKD-MBD needs to be further studied. Therefore, the aim of this research is to (1) explore the association between the increasing stages of CKD and the development of cortical PI; (2) judge the mediation effect of several bone metabolism markers (i.e., PTH, β-CTX, T-P1NP, OC, and 25OHD) and dual-energy X-ray absorptiometry (DXA) lumbar spine bone mineral density (BMD) on this association.

## Materials and Methods

### Subjects

This cross-sectional cohort study was prospective, which was approved by Medical Ethics Committee of Tongji Hospital of Huazhong University of Science and Technology, TJ-IRB20210108. We obtained written informed consent from every participant before the research. This trial was registered with ClinicalTrials.gov as NCT04564924. The subjects were recruited from September 1, 2020, to April 11, 2021. We recruited one hundred patients from the Department of Nephrology of Tongji Hospital. Three initially collected patients were excluded as a result of the replacement of coils, one patient was excluded because the tibia cortex was too thin to draw the ROI, and one patient was excluded due to poor image quality. The remaining 95 patients were included. All participants were over 18 years old and ambulatory. The specific diagnostic criteria for CKD are: (1) any evidence of kidney damage (pathological abnormalities, abnormal blood or urine components, or abnormal imaging tests.) or (2) eGFR < 60 mL/min/1.73m^2^ longer than 3 months. The inclusion criteria were hospitalized patients diagnosed with CKD stage 2-5. The reason why we did not include CKD stage 1 (eGFR > 90 mL/min/1.73m^2^) is (1) The onset of CKD is insidious in the ultra-early stage, and the clinical symptoms of CKD stage 1 patients are atypical and cannot be firmly diagnosed by eGFR alone. The evidence of kidney damage should be acquired. Fewer than 5% of early-stage CKD patients are aware of their disease (23). Therefore, there are few such patients in clinical practice; (2) In the process of recruiting patients, we hardly recruited patients with CKD stage 1; (3) Almost none studies on CKD-MBD in last decades have included CKD stage 1 patients (8, 15, 24, 25). Key exclusion criteria included any disease known to affect bone metabolism (e.g., diabetes, hyperthyroidism, rickets, osteomalacia, Paget’s disease, acromegaly, scurvy (vitamin C deficiency), history of malignant tumors, treatment with radiotherapy or chemotherapy, fractures within 6 months, lumbar or calf trauma surgery, rheumatic immunity disease, scoliosis, anorexia nervosa, and motor neuron disease); use of medications known to influence bone metabolism (e.g., bisphosphonates, oral glucocorticoids, steroid hormones, and salmon calcitonin); and general contraindications to MRI exam (e.g., IUD, pacemaker, cochlear implant, and claustrophobia).

### MRI Scanning

Studies were conducted on a 3 Tesla (T) clinical scanner (Signa Pioneer, GE Healthcare, USA) using a medium soft coil, and the dominant leg was scanned. A 3D double-echo UTE-Cones sequence (TR/TE_1_/TE_2_, 11.5/0.032/4.4 ms; FOV, 16 cm; slice thickness, 3 mm; flip angle, 9˚; bandwidth, 62.5 kHz; in-plane spatial resolution, 0.89mm*0.89mm; 12 axial images; and scan time, 54 s) was adopted to get tibial images. To locate the thickest part of the tibial cortex, in the positioning phase, the spine coil was used to image the full length of the tibia and was then positioned at the thickest plane (approximately in a 38% place of the tibial length close to the lateral malleolus) of the tibia in the coronal and sagittal planes.

The reason for tibial cortex used for our study is mainly due to two reasons: (1) In the study of bone pore water, the tibial cortex and the femoral neck cortex are often selected parts (8, 20, 22, 26, 27). (2) According to other research and our pre-experiment, compared with the femoral neck cortex, the tibial cortex is very thick, and the boundary with the bone marrow is very clear, which is more beneficial to accurately draw the ROI of bone cortex.

### Image Processing

**Figure 1:**
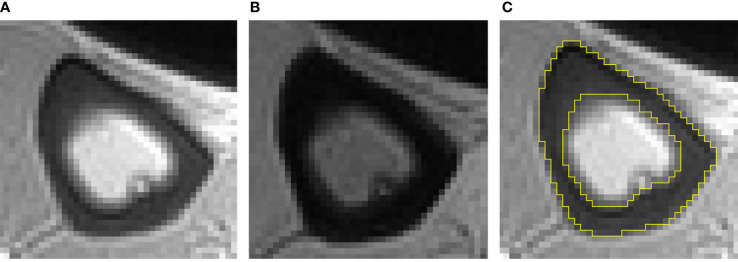
Typical double-echo UTE images and ROI of the tibia **(A–C)**. **(A)** The image of first echo; **(B)** the image of second echo; **(C)** example of ROI.

All images were processed with ImageJ (National Institutes of Health). Two experienced musculoskeletal radiologists (one had three years of experience and one had five years of experience), who were blinded to the subjects’ clinical data, independently drew the ROI. The ROI was drawn on TE_1_ and then copied to TE_2_. PI = TE_4.4_intensity/TE_0.032_intensity. The ROI was limited to a compacting cortex in appearance, and the trabecularized transition zone was not included, because most UTE signals in these parts were derived from adipose tissue, interfering with water signal of the cortical, thereby interfering with the measurement of PI. The tibial cortex was analyzed on the middle 5 layers of the 12 axial images. Subsequently, the PI value of each layer was calculated separately, and then the average value of PI was obtained. **Figure 1** shows examples of the ROI.

### Kidney Function

All subjects completed the kidney function examination within one week of the MRI examination. We used serum creatinine level to obtain the estimated glomerular filtration rate (eGFR) through the CKD-EPI formula (28). The stages of CKD were based on the eGFR.

### Bone Metabolism Markers

Bone metabolism markers, namely, PTH, β-CTX, T-P1NP, OC, and 25OHD, were assessed within one week of the MRI examination.

### Dual-Energy X-Ray Absorptiometry

A Prodigy Lunar scanner (GE Healthcare, Waukesha, WI, USA) was used to evaluate the areal BMD and T-score of the lumbar spine (from L1 to L4).

### Statistical Analysis

**Figure 2:**
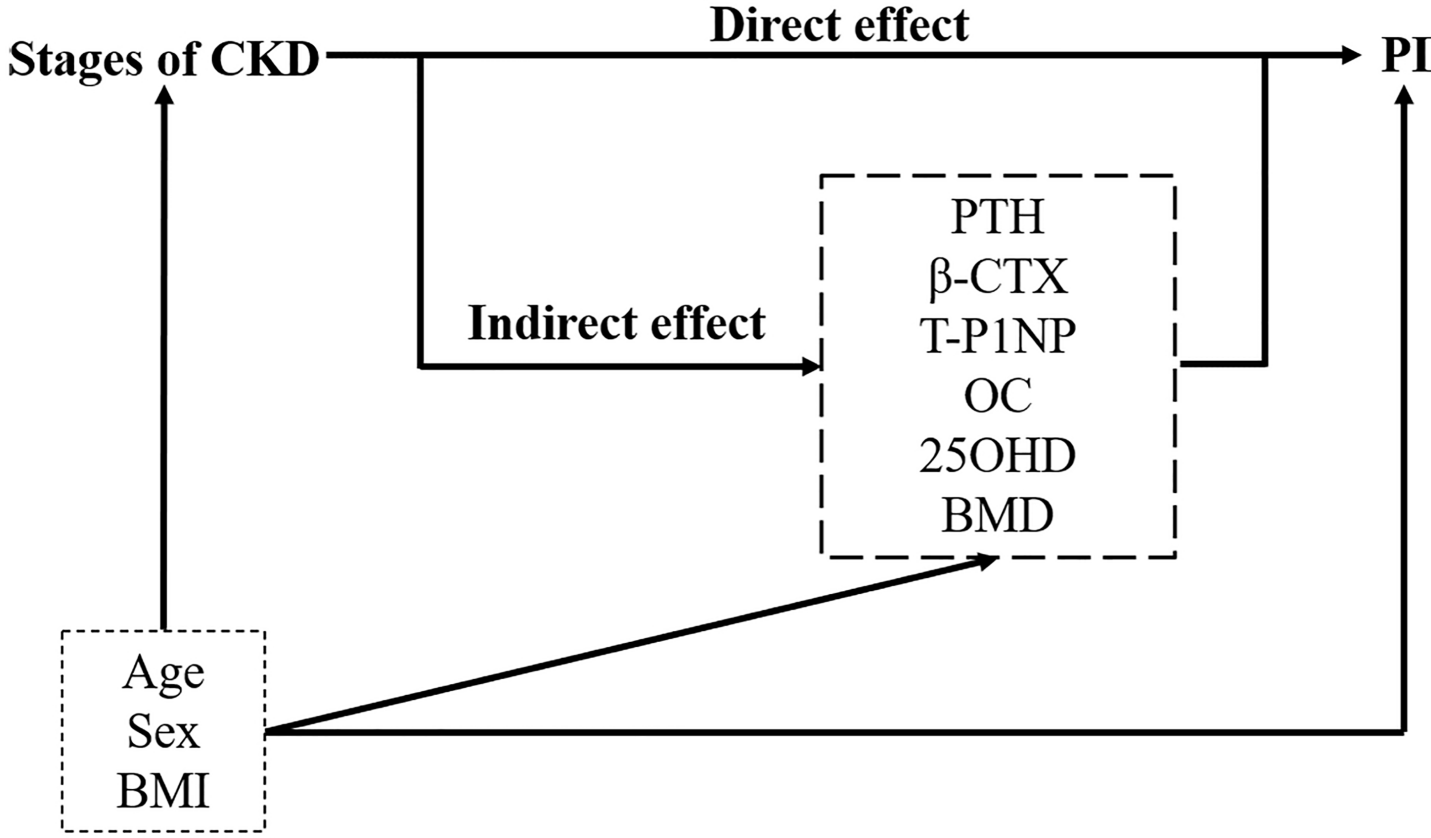
Path diagram of the mediation relationship between grouping (the stages of CKD), mediators (bone metabolism markers and lumbar BMD) and outcome (PI). Adjusting for confounders: age, sex, and BMI. Direct and indirect effects are obtained from multiple linear regression. PTH, parathyroid hormone; β-CTX, β-CrossLaps; T-P1NP, total procollagen type I amino-terminal propeptide; OC, osteocalcin; 25OHD, 25-hydroxyvitamin D; BMD, bone mineral density; PI, porosity index.

We summarized continuous variables as the mean ± SD, and categorical variables as frequency and percentage. Based on eGFR, patients were divided into four stages. The baseline characteristics between the four stages were compared for linear trends using one-way analysis of variance (ANOVA) for continuous variables and Chi-squared statistics for categorical variables. Partial correlation analysis was performed to address associations between PI, eGFR and potential mediators, controlling for age and BMI. Multiple linear regression models were used to assess the association between CKD stages and PI value. Crude effects were tested in an unadjusted model. Next model, we adjusted for age, sex, and BMI. In the final model, we introduced the potential mediators that were correlated with PI value in partial correlation analysis as well as the mediators’ interactions with CKD stages. We calculated *P* for the linear trend by analyzing CKD stages as a continuous instead of an ordinal variable. Then, a separate exploratory mediation analysis was carried out for each metabolic indicator to explore the impact of CKD stages and mediators on the PI value. The mediation R package (29) was used for mediation analysis. In this analysis, statistical models were simplified by treating CKD stages as interval variables. *P*-values and nonparametric bootstrap confidence intervals were estimated from one thousand simulations. **Figure 2** shows the tested hypothesized pathways in this study, which is the underlying model of this analysis. The model assumes that there is a causal relationship between the selected bone metabolism markers and the progression of CKD and cortical bone PI. The model includes age, sex, and BMI to adjust for potential confounding factors. Direct and indirect effects are obtained from multiple linear regression. The interobserver variability of PI was assessed between two independent raters using intraclass correlation coefficients (ICCs). The ICCs were computed by using the two-way mixed model for absolute agreement and single measures. An ICC value greater than 0.90 was considered excellent, and a value between 0.75 and 0.90 was considered good. All statistical analyses were performed in R v4.0.3. We set the statistical significance at *P* < 0.05.

## Results

### Baseline Characteristics

**Table 1** reports the baseline characteristics for the four CKD stages (12 people in stage 2, 23 people in stage 3, 20 people in stage 4, and 40 people in stage 5). The mean age, sex and BMI distributions were comparable across all four groups. Patients with more severe CKD had significantly higher intact PTH, β-CTX, T-P1NP, OC and PI values. No difference could be observed in 25OHD or BMD.

**Table 1:** Baseline characteristics by the stages of CKD.

	Total	Stages of CKD				*P*_trend_
		2	3	4	5	
n	95	12 (12.6)	23 (24.2)	20 (21.1)	40 (42.1)	na
Males (%)	48 (50.5)	9 (75)	10 (43.5)	12 (60)	17 (42.5)	0.158
Age (years)	49.7 (12.5)	52.9 (8.1)	49.8 (12.8)	49.5 (13.8)	48.7 (13.0)	0.360
BMI (kg/m^2^)	23.2 (3.4)	24.3 (4.2)	22.5 (3.3)	23.8 (3.7)	22.9 (2.9)	0.542
eGFR (mL/min/1.73m^2^)	27.2 (22.0)	71.0 (5.7)	41.1 (9.0)	23.3 (4.1)	8.1 (3.5)	**< 0.001**
intact PTH (pg/mL)	150.2 (124.7)	64.9 (51.2)	84.9 (54.5)	139.9 (81.5)	218.4 (149.2)	**< 0.001**
β-CTX (ng/mL)	1.23 (1.00)	0.58 (0.39)	0.75 (0.47)	0.91 (0.54)	1.86 (1.18)	**< 0.001**
T-P1NP (ng/mL)	122.7 (140.2)	29.8 (19.9)	40.6 (49.9)	88.6 (74.9)	214.9 (164.8)	**< 0.001**
OC (ng/mL)	73.9 (66.3)	27.2 (19.2)	30.4 (24.2)	58.6 (39.6)	120.5 (72.5)	**< 0.001**
25OHD (ng/mL)	18.5 (8.5)	21.9 (8.2)	19.8 (8.5)	16.8 (7.7)	17.6 (8.8)	0.102
L1-L4 BMD (g/cm^2^)	1.11 (0.16)	1.17 (0.14)	1.14 (0.17)	1.07 (0.15)	1.10 (0.16)	0.120
L1-L4 T-score	-0.83 (1.23)	-0.45 (1.07)	-0.59 (1.40)	-1.11 (1.02)	-0.93 (1.25)	0.151
PI (%)	20.2 (2.4)	18.2 (1.8)	19.8 (2.0)	20.2 (2.1)	21.1 (2.4)	**< 0.001**

Data are presented as means (standard deviation) and numbers (%). In Appropriate time, one-way ANOVA and Chi-square are used. Bold P_trend_ indicates statistical significance.

### Partial Correlation Between PI, eGFR and Potential Mediators

Significant correlations were revealed between the PI value and different bone metabolism markers (PTH: r = 0.381, *P* < 0.001; β-CTX: r = 0.375, *P* < 0.001; T-P1NP: r = 0.327, *P* = 0.001; OC: r = 0.357, *P* < 0.001). Similarly, significant correlations were revealed between the eGFR and different bone metabolism markers (PTH: r = -0.499, *P* < 0.001; β-CTX: r = -0.470, *P* < 0.001; T-P1NP: r = -0.512, *P* < 0.001; OC: r = -0.538, *P* < 0.001) (**Table 2**).

**Table 2:** Adjusted correlation coefficients between PI, eGFR and potential mediators.

	PI	eGFR
PTH	**0.381 (<0.001)**	**-0.499 (<0.001)**
β-CTX	**0.375 (<0.001)**	**-0.470 (<0.001)**
T-P1NP	**0.327 (0.001)**	**-0.512 (<0.001)**
OC	**0.357 (<0.001)**	**-0.538 (<0.001)**
25OHD	0.058 (0.578)	0.171 (0.101)
L1-L4 BMD	-0.139 (0.183)	0.227 (0.029)
L1-L4 T-score	-0.135 (0.198)	0.215 (0.039)

Partial correlation coefficients adjusted by age, and eGFR. Data are presented as means (P-value). The bold part indicates significant meaning.

### Multiple Linear Regression Analysis

In the crude model (model 1), we observed a positive association of CKD stages with PI, again with a significant gradient [stage 3 versus 2: β (95% CI) 1.58 (0.02, 3.14), stage 4 versus 2: β (95% CI) 2.01 (0.41, 3.61), stage 5 versus 2: β (95% CI) 2.93 (1.48, 4.37), *P*_trend_ < 0.001; In model 2, after adjusting for age, sex and BMI, the association was attenuated, but significance remained [stage 4 versus 2: β (95% CI) 1.96 (0.32, 3.59), stage 5 versus 2: β (95% CI) 2.83 (1.32, 4.33), *P*_trend_ < 0.001]. In model 3, after introducing all potential mediators (PTH, β-CTX, T-P1NP, and OC) that were correlated with PI value in partial correlation analysis as well as the mediators’ interactions with CKD stages, we found that the association between CKD stages and PI were no longer significant. (**Table 3**).

**Table 3:** Association of the stages of CKD with PI.

Outcome	Stages of CKD				*P*_trend_
	2	3	4	5	
Model 1	(ref.)	**1.58 (0.02, 3.14)**	**2.01 (0.41, 3.61)**	**2.93 (1.48, 4.37)**	**<0.001**
Model 2	(ref.)	1.49 (-0.13, 3.11)	**1.96 (0.32, 3.59)**	**2.83 (1.32, 4.33)**	**<0.001**
Model 3	(ref.)	1.35 (-0.25, 2.96)	1.53 (-0.12, 3.18)	1.73 (-0.02, 3.49)	0.084

Data are presented as regression coefficient (95% CI). The bold part indicates significant meaning.

Model 1: crude stages of CKD (Stage 2 is reference category).

Model 2: Model 1 + age, sex, and BMI.

Model 3: Model 2 + potential mediators (PTH, β-CTX, T-P1NP, and OC) (and their interactions with CKD stages).

### Mediation Analysis

**Figure 3:**
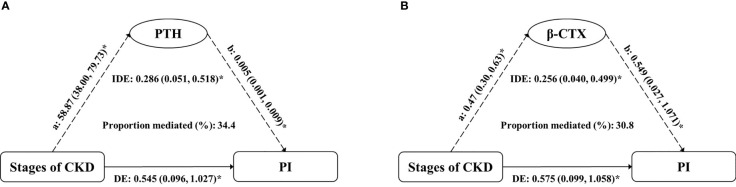
Estimates of the mediation effect of **(A)** PTH and **(B)** β-CTX about the association between the stages of CKD and PI value, after adjusting for age, sex and BMI. Note: **(A)** the effect of grouping on mediator; **(B)** the main effect of mediator on PI; IDE, indirect effect (Stages of CKD→PTH/β-CTX→PI); DE, direct effect (Stages of CKD→PI); PTH, parathyroid hormone; β-CTX, β-CrossLaps; PI, porosity index. *P < 0.05.

According to model 3, we found that the potential mediators were in the four bone metabolism markers (PTH, β-CTX, T-P1NP, and OC). At the same time, due to the multicollinearity between these four markers and their correlation with eGFR, in order to explore the impact of CKD stages and these four markers on PI value, we conducted simple causal mediation analysis for these four markers respectively. The main effects of PTH and β-CTX were significant (**Table S2**). We tested interactions of the stages of CKD with each potential mediator respectively (adjusted for age, sex and BMI); none of them was significant (*P* > 0.05). The association of CKD stages and PI mediated 34.4% (*P* = 0.012) and 30.8% (*P* = 0.024) of the total effect by increased PTH and β-CTX, respectively. No significant mediation was observed by T-P1NP and OC, 25(OH) vitamin D or BMD (**Table 4**). The results of mediation analysis of PTH and β-CTX were shown in **Figure 3**. After adjusting for confounding factors, one increase in CKD stage was associated with an increase of 58.87 pg/mL (95% CI: 38.00, 79.73) in PTH, and associated with an increase of 0.545% (95% CI: 0.096, 1.027) in PI; for every 1 pg/mL increase in PTH was associated with an increase of 0.005% (95% CI: 0.001, 0.009) in PI; the increased PTH mediated 34.4% of the total effect of CKD stages on PI. Similarly, one increase in CKD stage was associated with an increase of 0.47 ng/mL (95% CI: 0.30, 0.63) in β-CTX, and associated with an increase of 0.575% (95% CI: 0.099, 1.058) in PI; for every 1 ng/mL increase in β-CTX was associated with an increase of 0.549% (95% CI: 0.027, 1.071) in PI; the increased β-CTX mediated 30.8% of the total effect of CKD stages on PI. The association of CKD stages with potential mediators was shown in **Table S1**. The main effect of potential mediators was shown in **Table S2**.

**Table 4:** Mediators of the association between CKD stages and PI.

Mediators	IDE	*P*	DE	*P*	Proportion mediated	*P*
PTH	0.286 (0.051, 0.518)	**0.012**	0.545 (0.096, 1.027)	**0.012**	0.344 (0.051, 0.824)	**0.012**
β-CTX	0.256 (0.040, 0.499)	**0.024**	0.575 (0.099, 1.058)	**0.020**	0.308 (0.038, 0.795)	**0.024**
T-P1NP	0.198 (-0.064, 0.477)	0.140	0.633 (0.096, 1.191)	**0.022**	0.238 (-0.079, 0.794)	0.140
OC	0.242 (-0.081, 0.529)	0.114	0.589 (0.067, 1.168)	**0.024**	0.292 (-0.083, 0.862)	0.114

Data are presented as means (95% CI). Nonparametric bootstrap Confidence Intervals are estimated from 1000 simulations adjusted for age, sex, and BMI. Bold P-values indicate statistical significance. IDE, indirect effect; DE, direct effect.

### Interobserver Variability

The ICC was 0.85 (95% CI: 0.430, 0.942).

## Discussion

We examined the associations of CKD stages with the PI of the tibial cortex. More severe CKD is associated with higher PI values, independent of age, sex and BMI, while dependent of potential mediators. It was suggested that the association between CKD stages and tibial PI was partly due to PTH and β-CTX through exploratory mediation analysis.

Although the importance of cortical porosity for bone biomechanics has been increasingly appreciated (7, 30, 31), almost no one has assessed it noninvasively *in vivo*. Through PI measurements, we evaluated cortical porosity of the tibia by using double-echo UTE MRI sequences. The mean PI value of all subjects measured in our study was 20.2% ± 2.4%, which is consistent with the tibial cortical PI value of 20% ± 3.8% (range, 15%-31%) of 34 old menopausal women aged 55-80 years (20). This method can quantify the changes in cortical PI in CKD patients, providing a new choice to monitor and detect the deterioration of cortical bone microarchitecture in CKD patients.

The mechanisms underlying the association between CKD stages and cortical porosity are not completely clear, and few related studies have been conducted. Nickolas et al. (8) performed HR-pQCT scans on the distal tibia and distal radius. They found that dialysis was an independent factor in the increase in cortical porosity. On the basis of this research, our study further covered more complete CKD stages, supplemented the current evidence, and further showed that the progression of CKD is closely associated with increased cortical porosity. Nevertheless, we should not assume that more severe CKD necessarily corresponds to greater PI values, because the actual mechanism by which CKD drives cortical pore enlargement is still largely unknown (32). The factors leading to bone deterioration in patients with CKD are very complex and include the severity of hyperparathyroidism, the level of various bone metabolism markers, the degree of bone turnover and the impact of CKD itself on the bones, and these factors are interrelated (33–35). Consequently, we further conducted an exploratory mediation analysis. We tested each mediator separately with corrected age, sex and BMI to prevent overadjustment.

Among the indicators included in this study, high PTH was the strongest mediator. PTH can directly stimulate osteocytes to release receptor activator of nuclear factor κB ligand (RANKL), which results in increased osteoclast generation (36). In a study of CKD mice, Metzger et al. (37) found that PTH and cortical porosity increased with time, meanwhile RANKL increased and osteocyte apoptosis decreased. Therefore, they speculated that high PTH could prolong the life of osteocytes and lead to sustained RANKL and thus osteoclastic bone resorption in the cortex, promoting the cortical porosity formation. At present, PTH is still the most commonly used diagnostic aid for MBD. On the basis of the 2017 Kidney Disease: Improving Global Outcomes (KDIGO) guidelines, the level of bone turnover can be classified according to the level of PTH (2). Recently, some studies have shown that increased porosity is associated with increased PTH (3, 8, 26, 37, 38), which is consistent with our results. At the same time, our study also found that the increase in cortical porosity in people at different CKD stages is partly due to higher PTH.

Second, our research found that β-CTX was also a mediator. β-CTX is a marker of bone resorption, which can reflect osteocyte activity (39). At present, there is no consensus on the application of β-CTX in MBD. Some studies indicate that β-CTX is a bone turnover biomarker which exists direct release during bone resorption and could be used to more accurately assess the bone turnover in CKD (12). At the same time, Nishiyama et al. (24) found that higher levels of β-CTX and worsening cortical porosity have a linear dose-response relationship, which suggested that β-CTX could predict cortical deterioration. However, some studies suggested that β-CTX was not suitable as a diagnostic aid for MBD due to the influence of renal function (40). The role of this indicator in CKD-MBD needs further study. T-P1NP and OC did not show mediation effects in this study, possibly because these two indicators were greatly affected by renal clearance and could not accurately reflect bone deterioration in patients with CKD. There was also no mediation effect of 25OHD, which may due to supplement with vitamin D for the most patients with end-stage CKD, so that the 25OHD level in end-stage patients was higher than that in patients at earlier stages in our study. Due to the analytical and biological variability of these serum markers, there are few studies on bone metabolism markers other than PTH in CKD, and the clinical applications of these indicators are limited. The results of this study could provide some hints for further research in the future.

Interestingly, we did not observe a mediation effect of lumbar BMD measured by DXA, and we found that as CKD progressed, BMD did not continue to decrease. It might be that the BMD measured by DXA was a real BMD, which is not distinguishable between trabecular and cortical bone (11, 41), or that BMD itself has a small contribution to porosity. Because cortical and trabecular bone behave differently in answer to increased parathyroid activity (decrease and increase, respectively) and larger trabecular BMD can mask the loss of cortical bone, BMD measured by DXA in CKD patients may be higher, equal to or lower than that in healthy controls (13). Some studies were consistent with our study. Rajapakse et al. (20) showed that the femur and spine BMD measured by DXA had no significant correlation with PI. They also found that the cortical BMD obtained using pQCT imaging in bone specimens can merely partially explain the change in porosity, highlighting the need to estimate porosity without the dependence of BMD. Similarly, Carvalho et al. (42) found that there was no significant correlation between DXA parameters and cortical porosity for either internal cortical bone or external cortical bone.

The advantages of this study include: (1) The assessment of relatively complete indicators related to bone deterioration. (2) There is currently little evidence for indicators other than PTH in CKD, and this study can be used as a supplement to some extent. (3) We prospectively included CKD stages 2-5 patients in strict accordance with the inclusion criteria and exclusion criteria that may affect bone metabolism. (4) We also explored the mediation effect of a series of potential mediators, which can reflect the inherent causal relationship between CKD progress and the changes in PI values, which is not seen in the current CKD-MBD research. At the same time, this study has some limitations: (1) Many drugs and diseases can affect bone metabolism, so we excluded diseases and drugs that are known to affect bone metabolism as much as possible based on relevant research. Dialysis has been proved to be an independent factor of PI increase (8), but this study did not include dialysis patients. Our follow-up study will take dialysis patients as a separate group for further study. (2) Due to the limited detection level, we did not include TRAP5b or BSAP, two markers of bone metabolism that are considered to be independent of glomerular filtration rate.

In conclusion, the progression of CKD is associated with increased cortical PI values, and this association may be partly mediated by increased PTH and β-CTX. Our study provides a new idea to monitor bone health in patients with CKD, and reveal the internal mechanism of bone deterioration caused by CKD to some extent. Further work needs to be done to study other deep possible mechanisms about the association.

## Data Availability Statement

The raw data supporting the conclusions of this article will be made available by the authors, without undue reservation.

## Ethics Statement

The studies involving human participants were reviewed and approved by Medical Ethics Committee of Tongji Hospital, Tongji Medical College, Huazhong University of Science and Technology. The patients/participants provided their written informed consent to participate in this study.

## Author Contributions

FH and XL concept and design. DW, BH, and YL devised the outline of the manuscript. YX and TH designed the study and evaluated the data. YW, SH, and YZ collected the information and analyzed the data. YX wrote the manuscript. PZ and JL created the figures for the manuscript. WL and XL critically revised the manuscript. All authors contributed to the article and approved the submitted version.

## Funding

This study was supported by the National Natural Science Foundation of China (NSFC) (No. 31630025 and 81930045).

## Conflict of Interest

The authors declare that the research was conducted in the absence of any commercial or financial relationships that could be construed as a potential conflict of interest.

## Publisher’s Note

All claims expressed in this article are solely those of the authors and do not necessarily represent those of their affiliated organizations, or those of the publisher, the editors and the reviewers. Any product that may be evaluated in this article, or claim that may be made by its manufacturer, is not guaranteed or endorsed by the publisher.

## References

[1] Khairallah P Nickolas TL. Updates in CKD-Associated Osteoporosis. Curr Osteoporos Rep (2018) 16(6):712–23. doi: 10.1007/s11914-018-0491-3 PMC6264896 30353319

[2] Kidney Disease: Improving Global Outcomes (KDIGO) CKD-MBD Update Work Group. KDIGO 2017 Clinical Practice Guideline Update for the Diagnosis, Evaluation, Prevention, and Treatment of Chronic Kidney Disease-Mineral and Bone Disorder (CKD-MBD). Kidney Int Suppl (2011) (2017) 7(1):1–59. doi: 10.1016/j.kisu.2017.04.001 30675420 PMC6340919

[3] Sharma AK Toussaint ND Masterson R Holt SG Rajapakse CS Ebeling PR. Deterioration of Cortical Bone Microarchitecture: Critical Component of Renal Osteodystrophy Evaluation. Am J Nephrol (2018) 47(6):376–84. doi: 10.1159/000489671 29791896

[4] Pimentel A Ureña-Torres P Zillikens MC Bover J Cohen-Solal M. Fractures in Patients With CKD-Diagnosis, Treatment, and Prevention: A Review by Members of the European Calcified Tissue Society and the European Renal Association of Nephrology Dialysis and Transplantation. Kidney Int (2017) 92(6):1343–55. doi: 10.1016/j.kint.2017.07.021 28964571

[5] Moorthi RN Fadel W Eckert GJ Ponsler-Sipes K Moe SM Lin C. Bone Marrow Fat Is Increased in Chronic Kidney Disease by Magnetic Resonance Spectroscopy. Osteoporos Int (2015) 26(6):1801–7. doi: 10.1007/s00198-015-3064-7 PMC4582653 25701052

[6] Clarke B. Normal Bone Anatomy and Physiology. Clin J Am Soc Nephrol (2008) 3 Suppl 3(Suppl 3):S131–9. doi: 10.2215/CJN.04151206 PMC3152283 18988698

[7] Morgan EF Unnikrisnan GU Hussein AI. Bone Mechanical Properties in Healthy and Diseased States. Annu Rev BioMed Eng (2018) 20:119–43. doi: 10.1146/annurev-bioeng-062117-121139 PMC6053074 29865872

[8] Nickolas TL Stein EM Dworakowski E Nishiyama KK Komandah-Kosseh M Zhang CA. Rapid Cortical Bone Loss in Patients With Chronic Kidney Disease. J Bone Miner Res (2013) 28(8):1811–20. doi: 10.1002/jbmr.1916 PMC3720694 23456850

[9] Nickolas TL Leonard MB Shane E. Chronic Kidney Disease and Bone Fracture: A Growing Concern. Kidney Int (2008) 74(6):721–31. doi: 10.1038/ki.2008.264 PMC4139042 18563052

[10] Evenepoel P Cavalier E D'Haese PC. Biomarkers Predicting Bone Turnover in the Setting of CKD. Curr Osteoporos Rep (2017) 15(3):178–86. doi: 10.1007/s11914-017-0362-3 28429254

[11] Silva BC Costa AG Cusano NE Kousteni S Bilezikian JP. Catabolic and Anabolic Actions of Parathyroid Hormone on the Skeleton. J Endocrinol Invest (2011) 34(10):801–10. doi: 10.3275/7925 PMC4315330 21946081

[12] Salam S Gallagher O Gossiel F Paggiosi M Khwaja A Eastell R. Diagnostic Accuracy of Biomarkers and Imaging for Bone Turnover in Renal Osteodystrophy. J Am Soc Nephrol (2018) 29(5):1557–65. doi: 10.1681/ASN.2017050584 PMC5967779 29555831

[13] Leonard MB. A Structural Approach to Skeletal Fragility in Chronic Kidney Disease. Semin Nephrol (2009) 29(2):133–43. doi: 10.1016/j.semnephrol.2009.01.006 PMC2705768 19371804

[14] Moe SM Chen NX Newman CL Gattone VH 2nd Organ JM Chen X. A Comparison of Calcium to Zoledronic Acid for Improvement of Cortical Bone in an Animal Model of CKD. J Bone Miner Res (2014) 29(4):902–10. doi: 10.1002/jbmr.2089 PMC3940692 24038306

[15] Marques ID Araújo MJ Graciolli FG Reis LM Pereira RM Custódio MR. Biopsy vs. Peripheral Computed Tomography to Assess Bone Disease in CKD Patients On Dialysis: Differences and Similarities. Osteoporos Int (2017) 28(5):1675–83. doi: 10.1007/s00198-017-3956-9 28204954

[16] Link TM. Osteoporosis Imaging: State of the Art and Advanced Imaging. Radiology (2012) 263(1):3–17. doi: 10.1148/radiol.2631201201 22438439 PMC3309802

[17] Manhard MK Nyman JS Does MD. Advances in Imaging Approaches to Fracture Risk Evaluation. Transl Res (2017) 181:1–14. doi: 10.1016/j.trsl.2016.09.006 27816505 PMC5357194

[18] Chang EY Du J Chung CB. UTE Imaging in the Musculoskeletal System. J Magn Reson Imaging (2015) 41(4):870–83. doi: 10.1002/jmri.24713 PMC4297256 25045018

[19] Jerban S Lu X Dorthe EW Alenezi S Ma Y Kakos L. Correlations of Cortical Bone Microstructural and Mechanical Properties With Water Proton Fractions Obtained From Ultrashort Echo Time (UTE) MRI Tricomponent T2* Model. NMR BioMed (2020) 33(3):e4233. doi: 10.1002/nbm.4233 31820518 PMC7161421

[20] Rajapakse CS Bashoor-Zadeh M Li C Sun W Wright AC Wehrli FW. Volumetric Cortical Bone Porosity Assessment With MR Imaging: Validation and Clinical Feasibility. Radiology (2015) 276(2):526–35. doi: 10.1148/radiol.15141850 PMC4517853 26203710

[21] Seifert AC Wehrli SL Wehrli FW. Bi-Component T2 Analysis of Bound and Pore Bone Water Fractions Fails at High Field Strengths. NMR BioMed (2015) 28(7):861–72. doi: 10.1002/nbm.3305 PMC4478152 25981785

[22] Chen M Yuan H. Assessment of Porosity Index of the Femoral Neck and Tibia by 3D Ultra-Short Echo-Time MRI. J Magn Reson Imaging (2018) 47(3):820–8. doi: 10.1002/jmri.25782 28561910

[23] Chen TK Knicely DH Grams ME. Chronic Kidney Disease Diagnosis and Management: A Review. JAMA (2019) 322(13):1294–304. doi: 10.1001/jama.2019.14745 PMC7015670 31573641

[24] Nishiyama KK Pauchard Y Nikkel LE Iyer S Zhang C McMahon DJ. Longitudinal HR-pQCT and Image Registration Detects Endocortical Bone Loss in Kidney Transplantation Patients. J Bone Miner Res (2015) 30(3):554–61. doi: 10.1002/jbmr.2358 25213758

[25] Woods GN Ewing SK Sigurdsson S Kado DM Ix JH Hue TF. Chronic Kidney Disease Is Associated With Greater Bone Marrow Adiposity. J Bone Miner Res (2018) 33(12):2158–64. doi: 10.1002/jbmr.3562 PMC6702945 30075054

[26] Trombetti A Stoermann C Chevalley T Van Rietbergen B Herrmann FR Martin PY. Alterations of Bone Microstructure and Strength in End-Stage Renal Failure. Osteoporos Int (2013) 24(5):1721–32. doi: 10.1007/s00198-012-2133-4 23100118

[27] Lu X Jerban S Wan L Ma Y Jang H Le N. Three-Dimensional Ultrashort Echo Time Imaging With Tricomponent Analysis for Human Cortical Bone. Magn Reson Med (2019) 82(1):348–55. doi: 10.1002/mrm.27718 PMC6491227 30847989

[28] Levey AS Stevens LA Schmid CH Zhang YL Castro AF 3rd Feldman HI. A New Equation to Estimate Glomerular Filtration Rate. Ann Intern Med (2009) 150(9):604–12. doi: 10.7326/0003-4819-150-9-200905050-00006 PMC2763564 19414839

[29] Tingley D Yamamoto T Hirose K Keele L Imai K. Mediation: R Package for Causal Mediation Analysis. J Stat Software (2014) 59:1–38. doi: 10.18637/jss.v059.i05

[30] Sundh D Mellström D Nilsson M Karlsson M Ohlsson C Lorentzon M. Increased Cortical Porosity in Older Men With Fracture. J Bone Miner Res (2015) 30(9):1692–700. doi: 10.1002/jbmr.2509 25777580

[31] Cooper DM Kawalilak CE Harrison K Johnston BD Johnston JD. Cortical Bone Porosity: What Is It, Why Is It Important, and How Can We Detect it? Curr Osteoporos Rep (2016) 14(5):187–98. doi: 10.1007/s11914-016-0319-y 27623679

[32] Metzger CE Swallow EA Allen MR. Elevations in Cortical Porosity Occur Prior to Significant Rise in Serum Parathyroid Hormone in Young Female Mice With Adenine-Induced CKD. Calcif Tissue Int (2020) 106(4):392–400. doi: 10.1007/s00223-019-00642-w 31832725 PMC7422916

[33] Nickolas TL Cremers S Zhang A Thomas V Stein E Cohen A. Discriminants of Prevalent Fractures in Chronic Kidney Disease. J Am Soc Nephrol (2011) 22(8):1560–72. doi: 10.1681/ASN.2010121275 PMC3148711 21784896

[34] Allen MR Swallow EA Metzger CE. Kidney Disease and Bone: Changing the Way We Look at Skeletal Health. Curr Osteoporos Rep (2020) 18(3):242–6. doi: 10.1007/s11914-020-00580-9 PMC7395483 32193793

[35] Sprague SM Bellorin-Font E Jorgetti V Carvalho AB Malluche HH Ferreira A. Diagnostic Accuracy of Bone Turnover Markers and Bone Histology in Patients With CKD Treated by Dialysis. Am J Kidney Dis (2016) 67(4):559–66. doi: 10.1053/j.ajkd.2015.06.023 26321176

[36] Bellido T Saini V Pajevic PD. Effects of PTH on Osteocyte Function. Bone (2013) 54(2):250–7. doi: 10.1016/j.bone.2012.09.016 PMC3552098 23017659

[37] Metzger CE Swallow EA Stacy AJ Allen MR. Strain-Specific Alterations in the Skeletal Response to Adenine-Induced Chronic Kidney Disease Are Associated With Differences in Parathyroid Hormone Levels. Bone (2021) 148:115963. doi: 10.1016/j.bone.2021.115963 33878503 PMC8102422

[38] Araujo MJ Karohl C Elias RM Barreto FC Barreto DV Canziani ME. The Pitfall of Treating Low Bone Turnover: Effects on Cortical Porosity. Bone (2016) 91:75–80. doi: 10.1016/j.bone.2016.07.009 27424935

[39] Wheater G Elshahaly M Tuck SP Datta HK van Laar JM. The Clinical Utility of Bone Marker Measurements in Osteoporosis. J Transl Med (2013) 11:201. doi: 10.1186/1479-5876-11-201 23984630 PMC3765909

[40] Sharma AK Masterson R Holt SG Toussaint ND. Emerging Role of High-Resolution Imaging in the Detection of Renal Osteodystrophy. Nephrol (Carlton) (2016) 21(10):801–11. doi: 10.1111/nep.12790 27042945

[41] Duan Y De Luca V Seeman E. Parathyroid Hormone Deficiency and Excess: Similar Effects on Trabecular Bone But Differing Effects on Cortical Bone. J Clin Endocrinol Metab (1999) 84(2):718–22. doi: 10.1210/jcem.84.2.5498 10022443

[42] Catarina C Magalhães J Neto R Pereira L Branco P Adragão T. Cortical Bone Analysis in a Predialysis Population: A Comparison With a Dialysis Population. J Bone Miner Metab (2017) 35(5):513–21. doi: 10.1007/s00774-016-0781-8 27830383

