# Supplementary material for: CKD Stages, Bone Metabolism Markers, and Cortical Porosity Index: Associations and Mediation Effects Analysis
Source: Front Endocrinol (Lausanne). 2021 Nov 5;12:775066. doi: 10.3389/fendo.2021.775066 (PMC8602844; doi:10.3389/fendo.2021.775066)
Supplement: Supplementary file 1 [file Table_1.docx]

**Table S1.** Associations of the stages of CKD with potential mediators.

| Stages of CKD | Adjusted changes (95% CI) of potential mediators | | | |
| --- | --- | --- | --- | --- |
|  | PTH | β-CTX | T-P1NP | OC |
| One stage increment | 58.87 (38.00, 79.73)^b^ | 0.47 (0.30, 0.63)^b^ | 71.83 (49.07, 94.58)^b^ | 36.04 (25.76, 46.32)^b^ |
|  |  |  |  |  |
| 2 | ref. | ref. | ref. | ref. |
| 3 | 22.68 (-56.48, 101.83) | 0.15 (-0.47, 0.77) | 9.96 (-74.63, 94.56) | -0.87 (-38.78, 37.05) |
| 4 | 80.47 (0.71, 160.23)^a^ | 0.33 (-0.29, 0.96) | 62.00 (-23.24, 147.24) | 31.53 (-6.67, 69.74) |
| 5 | 159.51 (86.08, 232.93)^b^ | 1.26 (0.69, 1.84)^b^ | 187.13 (108.66, 265.60)^b^ | 90.80 (55.63, 125.97)^b^ |
| *P* for trend | <0.001 | <0.001 | <0.001 | <0.001 |

^a^ *P* < 0.05, ^b^ *P* < 0.001. Adjusted for age, sex, and BMI.

**Table S2.** Estimates of main effects of potential mediators on PI.

|  | Main effect | *P* |
| --- | --- | --- |
| PTH | 0.005 (0.001, 0.009) | **0.023** |
| β-CTX | 0.549 (0.027, 1.071) | **0.040** |
| T-P1NP | 0.003 (-0.001, 0.007) | 0.164 |
| OC | 0.007 (-0.002, 0.015) | 0.124 |

Data are presented as regression coefficient (95% CI). All models are adjusted for age, sex, and BMI. Bold *P*-values indicate statistical significance.
